# Towards classifying species in systems biology papers using text mining

**DOI:** 10.1186/1756-0500-4-32

**Published:** 2011-02-04

**Authors:** Qi Wei, Nigel Collier

**Affiliations:** 1 Department of Informatics, The Graduate University for Advanced Studies (Sokendai), 2-1-2 Hitotsubashi, Chiyoda-ku, Tokyo; 2 National Institute of Informatics, 2-1-2 Hitotsubashi, Chiyoda-ku, Tokyo

## Abstract

**Background:**

In recent years high throughput methods have led to a massive expansion in the free text literature on molecular biology. Automated text mining has developed as an application technology for formalizing this wealth of published results into structured database entries. However, database curation as a task is still largely done by hand, and although there have been many studies on automated approaches, problems remain in how to classify documents into top-level categories based on the type of organism being investigated. Here we present a comparative analysis of state of the art supervised models that are used to classify both abstracts and full text articles for three model organisms.

**Results:**

Ablation experiments were conducted on a large gold standard corpus of 10,000 abstracts and full papers containing data on three model organisms (fly, mouse and yeast). Among the eight learner models tested, the best model achieved an F-score of 97.1% for fly, 88.6% for mouse and 85.5% for yeast using a variety of features that included gene name, organism frequency, MeSH headings and term-species associations. We noted that term-species associations were particularly effective in improving classification performance. The benefit of using full text articles over abstracts was consistently observed across all three organisms.

**Conclusions:**

By comparing various learner algorithms and features we presented an optimized system that automatically detects the major focus organism in full text articles for fly, mouse and yeast. We believe the method will be extensible to other organism types.

## Background

In recent years high throughput methods have led to a massive expansion in the free text literature on molecular biology. Automated text mining has developed as an application technology for formalizing this wealth of results into structured database entries. As has been well reported, unstructured knowledge in free texts is inconvenient and hard to share, organize, and acquire. The use of databases as stores of knowledge has made it much easier for biologists and other life scientists to keep up to date with new discoveries. Yeh et al. [1] outline two purposes for databases. The first one is as a place for experts to consolidate data, often including DNA sequence information, about a single organism or a single class of organisms. The second is to make the information searchable by using a variety of automated techniques. Biological experiments are yielding more and more results that can be formalized by registering them in databases such as MGD (Mouse Genome Database) [2], FlyBase [3], DictyDb [4], and Wormpep [5]. The curation of literature in databases is a skilled human task that ensures the data stored in them accurately reflects scientific fact. In particular, database curation in the life sciences helps to ensure data quality to enable quick access to the latest experimental results. The bottleneck is that curation is a time-consuming task requiring a high degree of skill. For example, MGD curators have to ensure that the stored publication data can be used to validate expressions of genes under certain conditions. In this paper we present a method of text classification to support database curators in the initial stages of their work by selecting full articles according to the main focus species. The value in this study is to present experimental evidence on the best models and features for this purpose.

In recognition of the growing importance of database curation a number of communities have become established to support development of gold-standard shared tasks. The Knowledge Discovery and Data Mining (KDD) Challenge CUP task in 2002 [1] focused on automating the work of curating Flybase, by identifying papers on the topic of gene expression in drosophila. The goal of the BioCreative [6] challenge was to pose tasks that would result in scalable systems for use by biology researchers and end users such as annotation database curators. BioCreative tried to address the database curation task by challenging participants to identify papers according to the evidence they contained for assigning GO codes to human proteins. The highest achieved F-score was 81% for human in BioCreative II reflecting the fact that this task still remains a very challenging one. The TREC Genomics [7] track featured a text categorization task in 2005 and 2006 with the best system achieving an F-score of 58.7%. Documents were classified according to how their content could be of help in assigning GO annotations to mouse genes.

Despite the relative successes of the above studies, a fundamental problem remains: how to classify texts into different types of model organism efficiently. In this paper, we present a system to classify full journal papers according to the main organism used in the experiment. A few previous studies such as Lin 2009 [8] have indicated the benefits of using full papers over abstracts for information extraction tasks; our experiments provide additional evidence to support this. Additionally we show the advantage of using species-gene association features with classification performance improving by 10%.

Text classification of full papers aims at automatically determining whether a paper belongs to one or more specific topic categories based on the contents described in the document. A species classification system would be especially valuable to database curators whose job is to review many documents and collect those containing certain experimental results pertaining to a specific organism. In earlier work, Liu and Wu (2004) [9] studied text classification for four organisms (fly, mouse, yeast and worm) using Medline abstracts, where the dataset had low levels of ambiguity between organisms (1%). They showed a best F-score around 94.1%. Rinaldi et al. [10] showed that in the BioCreative II corpus, the major organisms mentioned in full texts were humans (56.3%), mice (9.3%), yeast (6.5%) and C. elegans (6%). They devised a system that extracted a ranked list of species for each full paper texts and showed that such a list was good for disambiguation; the number of possible gene references was reduced to 45012 (p = 0.0308, r = 0.5763) from the initial annotation step 283556 (p = 0.0072, r = 0.7469). Wang and Matthews [11] created a rule-based system that used a combination of species name and gene name in the same sentence. They showed an 11.6 point improvement in F-score in classification by combining the rule-based system to the maxent classifiers. In our experiments, we explored similar features as a baseline and expand the investigation to include several new feature types such as species-gene proximity and species weight on eight learner models.

Many researchers consider text classification to be the first step in database curation. Yeh et al. [1] classified papers from the FlyBase dataset and determined whether the paper should be curated or not on the basis of the presence of experimental evidence for fly gene products, achieving a highest performance level of 76% using an information extraction approach with manually constructed rules. Donaldson et al. [12] used a support vector machine trained on the words in Medline abstracts to distinguish abstracts containing information on protein-protein interactions to help in curation of the BIND database; they got an F-score of 92%.

In our experiments reported below, we focus on classifying documents for three different organisms: fly, mouse and yeast. We believe this study contributes to the work on biological text classification and database curation and will aid in the task of gene name disambiguation.

## Methods

### Dataset

The dataset we employ was based on the BioCreative I task 1B corpus which was manually selected from three model organism databases: Fly [3] (*Drosophila melanogaster*), Mouse [2] (*Saccharomyces cerevisiae*), Yeast [13] (*Mus musculus*). PubMed IDs were selected from the databases and Medline abstracts were selected according to these PubMed identifiers to make up the BioCreative I task 1B corpus. There are 4 gene mentions in each abstract on average. We manually collected the corresponding full papers for the abstracts from PubMed and Google search. The final corpus contained 3761, 3572, 3725 papers for fly, yeast and mouse respectively.

### Work flow

**Figure 1:**
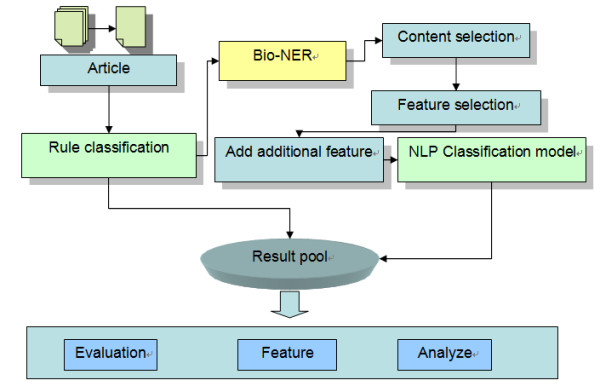
Experimental workflow

The workflow for the experiment is shown in Figure 1. (1) Documents were cleaned and saved in a standard format; (2) Documents were then classified using a rule-based classification model. The purpose of this step was to choose the easiest cases in the dataset and classify them first. The heuristic rule was simple: if a title contained only one organism mention then the text was tagged according to that organism. In this way 5% of documents were classified, and the remaining documents were resolved in the following steps; (3) AbGene [14] was used to annotate the gene names in each document and which part of the document should be used was determined by using a content selection model. One-hundred articles with similar structures (abstract, introduction, result, experiment, discussion, and conclusion) were selected manually and a gene-section distribution for these 100 articles was created. Based on this analysis the abstract, introduction, result and conclusion sections were selected, and other sections were excluded. If an article contained no significant sub-title to show these four sections, the gene distribution was counted and compared to the gold-standard gene distribution and the four sections were selected according to the similarity calculation. Gene names were selected as features. (4) Additional features such as title and journal name were then added; (5) Eight supervised models were used to classify the documents. In this step, the data remaining undecided from step (2) were used. We then analyzed the model's performance using ablation experiments on various combinations of features.

### Models

In our experiments, we compared eight supervised classification methods: Naïve Bayes, Conditional Random Fields, support vector machines (SVMs), Decision table, Decision trees, Logistics Regression as well as Adaboost and Bagging on the best performing models.

The Naïve Bayes model is a simple probabilistic classifier based on Bayes's theorem with strong independence assumptions that is widely used in text classification. The Naïve Bayes implementation we used was included in the Weka toolkit [15], default parameters were used for training.

Conditional random fields (CRF) [16] is a discriminative probabilistic framework that is used for labelling and segmenting sequential data. A CRF is an undirected graphical model that defines a single log-linear distribution over labelled sequences given a particular observation sequence. Recently Hirohata et al. [17] showed success in applying CRF for a document classification task. We applied the same broad methodology as Hirohata et al. in our implementation. We formulated the document classification task as a sequence labelling task by firstly labelling each document section with its focus species and then labelling the focus species for the whole document based on the sequence of section labels. The CRF++ toolkit [18] was used. The hyper-parameter to set the trade-off between over-fitting and under-fitting was set at 10. Default values were used for the other parameters.

SVMs were introduced by Vapnik [19] in 1995 as a learning system that uses a hypothesis space of linear functions in a high dimensional feature space, trained with a learning algorithm from optimization theory that implements a learning bias derived from statistical learning theory.

Boosting [20] and bagging [21] are generic methods aimed at aggregating classifiers for improved prediction performance using sample weighting and re-sampling respectively on the original training data. Both techniques can be applied to a variety of base learners and have been shown to give substantial gains in accuracy for classification tasks. In our experiments Naive Bayes was chosen as the base learner for its high level of performance in the stand alone task.

Decision tables [22] contains two major components, a list of attributes and a set of labelled instances on those attributes. Labelling is done by default on majority class matching and then by arbitrary tie breaking and attribute elimination. They have a close relation to rule-based knowledge bases. Decision trees [23] are potentially powerful predictors and explicitly represent the structure of a rule set in tree form with leaf nodes functioning as classification decisions and transitions along branches taking place according to attribute values. Logistic regress [24] is a popular discriminative classifier for modelling binary data. In our experiment, AdaBoost, Bagging, Decision tables, Decision trees and Logistic regress were implemented from the Weka toolkit.

For the named entity recogniser, AbGene was used to annotate the gene names in the document. AbGene [25] was pre-trained on annotated Medline abstracts with a reported F-score of 98%. Tanabe [14] showed that it is possible to use AbGene on full text articles from PubMed Central (PMC) with a reduced level of performance at 72.6% precision and 66.7% recall. Since our abstracts were selected from Medline and the full text was selected from PMC and Google search, we can expect broadly similar levels of performance with this earlier experiment.

### Features

The experiment tested several linguistic features which we describe in detail below:

(1) GN: Gene name terms

Following gene name annotation with AbGene, genes were listed according to their frequency in the document and the top n genes were selected as features to train the model. Here, n is a fixed number decided before the experiment. We varied n from 1 to 100 in preliminary experiments, with the results indicating that the larger n was, the better the results were. As n > 100 was difficult to handle using our CRF software due to machine memory limitations, n = 100 was used in the experiment.

(2) OF: Organism frequency

Organism name mentions were used as a reference for classifying the text into different model organisms. The organism names included not only mice, fly and yeast but also synonym words such as mouse, drosophila, and saccharomyces. This list was compiled by hand according to the NCBI taxonomy.

(3) MH: MeSH headings

Bloehdorn and Hotho [26] report that MeSH headings improved the accuracy of classification by 3% to 5%. We therefore selected the three frequently mentioned MeSH headings for each based on frequency in the training data.

(4) DT: Document title terms

Some of the document titles contained organism name mentions and gene name mentions which were then used as features in the rule classification model and NLP classification model.

(5) TS: Term-species

If one sentence contained a gene name and a species name, the weight of the species name was counted by using the distance between the species name and gene name. The total weight was tallied for each article, and the weight of the species name was used as a feature.

(6) JN: Journal Name

This was the name of the journal in which the abstract or article was published.

(7) NT: Number of terms

First, gene list was extracted from the training corpus and sorted by the frequency of the gene. Then the number of genes in the top-100 frequent gene list was counted.

(8) AGN: Additional gene name terms

When there was a gene-species pair in one sentence, the gene name and species name was used to find an additional gene name in UniProt. For example, there was a gene named "IL2", by looking up in UniProt, the additional gene name was "Interleukin".

### Evaluation metrics

We consider a binary label where one entity can be either positive (+) or negative (-). In Table 1, the label stands for its gold standard label, and the assignment stands for the label given by the model. TP stands for true positive, TN stands for true negative, FP stands for false positive, and FN stands for false negative. Standard evaluation measures are defined on the basis of these labels as follows:

**Table 1:** Scoring Matrix

		Assignment	
		
		+	-
Gold standard	+	TP	FN
	
	-	FP	TN

1. Precision P = TP/(TP+FP)

2. Recall: R = TP/(TP+FN)

3. F-score F = (2PR)/(P+R)

## Results

### Experiment one: Comparison on different learner models

In the first experiment, eight different models were selected: Naïve Bayes, AdaBoost, Bagging, Decision table, Decision tree, Logistics Regression, CRF and SVMs. Table 2 compares the 10-fold cross evaluation of the different models. NB had the highest F-score (84.8% for fly, 73.9% for mouse and 73.8% for yeast), and CRF had the second highest (80.2% for fly, 73.0% for mouse and 72.3% for yeast). AdaBoost and Bagging both used Naïve Bayes as the base learner, but we did not observe a significant improvement when using the basic feature set. Logistics Regress performed well on fly (79.6%) but not so well on the other two species. SVMs gave high precision but low recall in fly and yeast; high recall but low precision in mouse.

**Table 2:** Result of experiment one: comparison of different models

		F1			F1-JN			F1-MH		
		
		P	R	F	P	R	F	P	R	F
	fly	0.780	0.929	0.848	0.685	0.890	0.777	0.530	0.808	0.640
NB	mouse	0.810	0.680	0.739	0.697	0.620	0.656	0.683	0.410	0.776
	yeast	0.750	0.727	0.738	0.646	0.515	0.573	0.747	0.657	0.828

	fly	0.780	0.929	0.848	0.659	0.899	0.761	0.638	0.677	0.657
AdaBoost	mouse	0.810	0.680	0.739	0.697	0.620	0.656	0.696	0.550	0.615
	yeast	0.750	0.727	0.738	0.649	0.485	0.555	0.640	0.737	0.685

	fly	0.791	0.919	0.850	0.729	0.869	0.793	0.606	0.838	0.703
Bagging	mouse	0.788	0.670	0.724	0.670	0.670	0.670	0.831	0.490	0.616
	yeast	0.768	0.768	0.765	0.638	0.515	0.570	0.696	0.717	0.706

	fly	0.532	0.667	0.592	0.556	0.354	0.432	0.532	0.667	0.592
Decision Table	mouse	0.515	0.520	0.517	0.388	0.870	0.537	0.515	0.520	0.517
	yeast	0.740	0.545	0.628	0.727	0.081	0.145	0.740	0.545	0.628

	fly	0.637	0.798	0.709	0.500	0.687	0.579	0.341	0.606	0.436
Decision tree	mouse	0.557	0.640	0.595	0.500	0.550	0.524	0.339	0.400	0.367
	yeast	0.729	0.434	0.544	0.596	0.313	0.411	1.000	0.040	0.078

	fly	0.878	0.727	0.796	0.932	0.697	0.798	0.663	0.576	0.603
Logistics Regression	mouse	0.586	0.750	0.658	0.721	0.490	0.583	0.541	0.730	0.621
	yeast	0.705	0.626	0.663	0.513	0.808	0.627	0.740	0.545	0.628

	fly	0.762	0.868	0.802	0.688	0.879	0.764	0.503	0.830	0.621
CRF	mouse	0.789	0.700	0.730	0.725	0.640	0.669	0.766	0.390	0.511
	yeast	0.734	0.725	0.723	0.652	0.566	0.598	0.744	0.650	0.685

	fly	0.925	0.641	0.757	0.619	0.684	0.650	0.200	0.240	0.217
SVM	mouse	0.403	1.000	0.574	0.309	0.406	0.351	0.250	0.147	0.185
	yeast	0.636	0.194	0.297	0.400	0.207	0.273	0.356	1.000	0.525

F1: basic features used to train the model were GN, MH, DT, and JN.

F1-JN: the features used were GN, MH and DT.

F1-MH: the features used were GN, DT, and JN.

The model comparison used only the basic feature set (MeSH headings, journal name, gene name, and article title). We also did feature analysis on MeSH headings and journal name in this experiment. The analysis showed that by using MeSH headings as a feature, a 2% improvement in F-score was achieved by using Naïve Bayes and CRFs. The journal name feature improved the F-score by 1% by using Naïve Bayes and CRFs.

### Experiment two: Comparison of different feature sets

NB and CRF were selected as the two best performing models from Experiment one. This time we used an extended set of features that included TS (term-species) and OF (organism frequency) in 10-fold cross evaluation experiments. The best performing combination achieved an average F-score of 90.7%. As shown in Table 3, classification for fly achieved the best among the three kinds of organisms (97.1%) followed by mouse (88.6%) and yeast (85.5%). We considered that the reason for this is that for fly focussed experimental papers, the gene-species pairing gave a clear signal, whereas in mouse the organism was often considered as the experiment model for human so the gene-species pair and organism frequency became highly ambiguous. In yeast the species name of yeast was rarely mentioned in the paper. The most significant result was that by using TS, OF and AGN features; an improvement of 10% was achieved.

**Table 3:** Result of experiment two: comparison of different feature sets

		F1			F1+TS			F1+OF		
		
		P	R	F	P	R	F	P	R	F
	fly	0.78	0.929	0.848	0.97	0.97	0.97	0.792	0.931	0.856
NB	mouse	0.81	0.68	0.739	0.826	0.95	0.884	0.821	0.685	0.747
	yeast	0.75	0.727	0.738	0.929	0.788	0.852	0.762	0.731	0.746

	fly	0.762	0.868	0.802	0.965	0.952	0.958	0.771	0.87	0.818
CRF	mouse	0.789	0.7	0.73	0.814	0.878	0.845	0.791	0.71	0.748
	yeast	0.734	0.725	0.723	0.902	0.786	0.84	0.739	0.73	0.734

		**F1+NT**			**F1+ADN**			**F1+TS+OF+NT+ADN**		
		
		P	R	F	P	R	F	P	R	F

	fly	0.775	0.825	0.799	0.812	0.931	0.867	0.971	0.972	0.971
NB	mouse	0.823	0.621	0.708	0.823	0.712	0.763	0.827	0.953	0.886
	yeast	0.752	0.723	0.737	0.786	0.987	0.875	0.931	0.791	0.855

	fly	0.753	0.877	0.81	0.773	0.887	0.826	0.966	0.954	0.96
CRF	mouse	0.865	0.698	0.773	0.792	0.714	0.751	0.817	0.878	0.846
	yeast	0.729	0.727	0.728	0.762	0.751	0.756	0.901	0.788	0.841

F1: basic features used to train the model were GN, MH, DT, JN.

### Experiment three: Comparison on full texts and abstracts

Large-scale collections of abstracts are often used in life science classification experiments, whereas full text articles are rarely used due to difficulties in sourcing them from publishers and converting them into plain text format. This trend is now changing due to the availability of open source publications. However, the highly detailed experimental information contained in full text papers reveals new challenges for biomedical document classification. For example, Tanabe [14] showed that entities like restriction enzyme sites, laboratory protocol kits, primers, vectors, molecular biology supply companies, and chemical reagents are rarely mentioned in abstracts, but plentiful in the methods section of the full article. Their appearance adds to the previously mentioned morphological, syntactic and semantic ambiguities. To mitigate this issue, content selection was applied to filter data in the full articles according to sections. Secondly, the full text, especially the Method and Introduction sections, contain larger numbers of associated gene/protein mentions in comparison with the abstracts. Again, this can be partially mitigated by content selection.

On the other hand, there are also some advantages to using full texts over abstracts. Potential redundancy of information allows models with lower levels of recall to have several chances to discover reported facts such as the species-gene/protein features that we observed to be highly valuable when making decisions about focus species.

To confirm the value of using full texts we compared classification performance of the full texts from our corpus of abstracts to the original abstracts. The comparison is shown in Table 4. We performed a two tailed paired sample t-test to show that there is an improvement of 11 points in F-score. In these experiments 10 × 10 cross validation was used in conjunction with two-tailed corrected resample t-test (p < 0.001) as presented by Bouckaert and Frank 2004 [27].

**Table 4:** Comparison of full papers and abstracts

		full text(F1+TS+RN+NT+AND)			abstract (F1+TS+RN+NT+AND)		
		
		P	R	F	P	R	F
	Fly	0.971	0.972	0.971	0.812	0.892	0.850
NB	Mouse	0.827	0.953	0.886	0.755	0.763	0.759
	Yeast	0.931	0.791	0.855	0.791	0.748	0.769

	fly	0.966	0.954	0.960	0.820	0.898	0.857
CRF	mouse	0.817	0.878	0.846	0.732	0.741	0.736
	yeast	0.901	0.788	0.841	0.757	0.750	0.753

## Discussion

### Content selection

As discussed above, one difficulty for focus species classification on full text articles is that of content selection. Deciding which part of the document is the most valuable and developing a strategy to select it is quite a difficult issue given that documents in our collection come from different journals which have different section structures. As a proxy for explicit section headings we decided to use the gene mention distribution as a clue for partitioning the full text papers. However, this approach proven weak in cases where the test document contained more sections than the standard one (four sections mentioned in the methods). During analysis we found that using such section selections showed no improvement in F-score.

### Feature selection

Another challenge was feature selection. Rinaldi et al. [10] used the species name appearing in a document as a clue to find the correct topic organism. Our experiment built on Rinaldi's findings in that not only did it use the species word itself as a feature, it also used species-gene pairs appearing together in one sentence and weighted the species according to the distance between the gene and species. Doing so improved the average F-score by 12% compared to that for the basic feature set. Compared with Rinaldi's work, our approach showed an average 3% improvement in the F-score.

### Difficult case: multi-species mentioned in one paper

Although many researchers have focused on text classification in biology, their experiments have mainly been targeted at extracting information about single organisms. Considering the task in the real world; texts are often not clean data on specific organisms.

The most difficult cases we encountered were when the text contained multiple species names. As the abstract below (PMID: 11018518) illustrates, four kinds of species were mentioned: fly (*Drosophila melanogaster*), mouse, zebrafish and silkworm (*Bombyx mori*).

Coatomer is a major component of COPI vesicles and consists of seven subunits. The gamma-COP subunit of the coatomer is believed to mediate the binding to the cytoplasmic dilysine motifs of membrane proteins. We characterized cDNAs for Copg genes encoding gamma-COP from mouse, zebrafish, Drosophila melanogaster and Bombyx mori. Two copies of Copg genes are present in vertebrates and in B. mori. Phylogenetic analysis revealed that two paralogous genes had been derived from a single ancestral gene by duplication independently in vertebrates and in B. mori. Mouse Copg1 showed ubiquitous expression with the highest level in testis. Zebrafish copg2 was biallelically expressed in hybrid larvae in contrast to its mammalian ortholog expressed in a parent-of-origin-specific manner. A phylogenetic analysis with partial plant cDNA sequences suggested that copg gene was also duplicated in the grass family (Poaceae).

This is a special case, but approximately 5% of articles in our collection reported multiple species. In the future we will need to consider how to handle these special cases more efficiently.

## Conclusion

In this paper, we presented a system that automatically categorizes full text documents into three organism categories: mouse, fly and yeast. Eight different models were compared and different feature sets were tested in the experiment indicating the key importance of the term-species distance feature we introduced. We also compared full texts and abstracts and showed the benefit of full texts in this task. Although the experiment was undertaken on only three focus species, we believe the methods employed will be extensible to other organisms.

## Competing interests

The authors declare that they have no competing interests.

## Authors' contributions

QW conceived of the study and conducted the experiment. NC provided technical support for the data analysis and directed the work. Both authors contributed during the whole length of the project and writing of the paper.
